# Metabolic Regulation of Hypoxia-Inducible Factors in Hypothalamus

**DOI:** 10.3389/fendo.2021.650284

**Published:** 2021-03-08

**Authors:** Dan Du, Yugang Zhang, Canjun Zhu, Hong Chen, Jia Sun

**Affiliations:** ^1^Department of Endocrinology, Zhujiang Hospital, Southern Medical University, Guangzhou, China; ^2^Guangdong Province Key Laboratory of Animal Nutritional Regulation, College of Animal Science, South China Agricultural University, Guangzhou, China

**Keywords:** energy homeostasis, obesity, hypoxia-inducible factor (HIF), hypothalamus, proopiomelanocortin (POMC)

## Abstract

The earliest hypoxia-inducible factor (HIF) function was to respond to hypoxia or hypoxic conditions as a transcription factor. Recent studies have expanded our understanding of HIF, and a large amount of evidence indicates that HIF has an essential effect on central regulation of metabolism. The central nervous system’s response to glucose, inflammation, and hormones’ main influence on systemic metabolism are all regulated by HIF to varying degrees. In the hypothalamus, HIF mostly plays a role in inhibiting energy uptake and promoting energy expenditure, which depends not only on the single effect of HIF or a single part of the hypothalamus. In this paper, we summarize the recent progress in the central regulation of metabolism, describe in detail the role of HIF in various functions of the hypothalamus and related molecular mechanisms, and reveal that HIF is deeply involved in hypothalamic-mediated metabolic regulation.

## Introduction

The central nervous system (CNS) receives many peripheral signals, including nutrient signals, hormone and gastric vagal afferent signals transmit and integrate peripheral energy information through a complex neural network, regulating peripheral target organs such as adipose tissue, through the nerve-body fluid pathway. The hypothalamus, a part of CNS, contains essential nuclei that function as neuroendocrine cells. It is a key regulator of systemic metabolic homeostasis because it can combine nutritional information with hormonal signals and regulate food intake as well as peripheral metabolism according to energy utilization. Glucose, leptin, insulin, and orexin play a special role in the brain, especially in the hypothalamus. These signals ultimately affect the metabolic capacity or electrical excitability of hypothalamic neurons, thereby regulating the whole body through the hypothalamus.

The metabolism of neurons is closely related to oxygen sensing. Different oxygen concentration will lead to different metabolic states. HIF is a major transcription factor that responds to hypoxia and induces or suppresses genes. HIF is a diploid, which consists of a stable α—subunit and a constitutively expressed beta subunit. There are three subtypes of HIF-α, called HIF-1α, HIF-2α, and HIF-3α (1). There is little research on HIF-3α, but HIF-1α and HIF-2α are described in detail. HIF-1α and HIF-2α are similar in structure, but the former is generally expressed *in vivo*, while the latter is more cell-specific, they both express in the brain. When oxygen is sufficient, the E3 ubiquitin ligase, known as von Hippel Lindau disease tumor suppressor (pVHL), mediates the binding of α—subunit to ubiquitin and causes α—subunit to be degraded by the proteasome (1). Besides, the prolyl hydroxylase domain (PhD) can inhibit HIF-1α through the combination of targeting degradation and transcriptional inhibition under normoxic conditions (2). These two factors are regarded as the most common regulatory factors of HIF. But many metabolic factors such as glucose (3) and lipid (4–6), also affect the stability and expression of HIF. Functions of HIF are diverse in different organs. For example, overexpression of HIF-1α and HIF-2α is demonstrated to increase hepatic steatosis (7).

Meanwhile, ablation of intestine-specific Hif-2α can reverse high-fat diet (HFD)-induced obesity (2). As for cancer, HIF can promote the growth, invasion and metastasis of cancer cells such as breast cancer (8) and can help the pancreatic cancers metabolize glucose at higher rates that benefit their survival (9). However, HIF is not always a harmful factor in the human body, as it’s a crucial protein in CNS and participates in controlling homeostasis of metabolism. The importance of HIF-1α in regulating body weight, liver metabolism and glucose homeostasis is evident (4). It is also pointed out that HIF-2α can affect the energy balance to a certain extent (10). Based on all the available evidences that we will mention below, we can conclude that HIF plays a vital role in hypothalamus-mediated systemic metabolic regulation. In this case, it is of great significance to understand the characteristics of HIF to provide a new and more robust method for the treatment of metabolic diseases. This article reviews the roles of HIF in the hypothalamus-mediated regulation of metabolism.

## Hypoxia-Inducible Factors Induced Increase of Glucosensing and Glucose Metabolism in the Hypothalamus

Previous studies have shown that HIF deeply takes part in response to hypoxia and inflammation. However, more and more recent studies have shown that HIF can not only regulate its targeted cells but also regulate the metabolic activities of cells in other parts of the body in an indirect but profound way. For example, HIF has been shown to regulate glucose sensing in CNS. As one of the crucial nutritional sensing functions of the central nervous system, glucose sensing should not be ignored when considering metabolic disorders. Both the glucose from food and the glucose produced by the body should be adjusted correctly to maintain balance. It is necessary to study the regulation of glucose sensing and metabolism by HIF *via* CNS.

Unlike the peripheral response of pancreatic cells as the leading way to regulate blood glucose, the hypothalamus is an important part of glucose sensing (11). The process of hypothalamic neurons sensing glucose is called hypothalamic glucose sensing (HGS). Compared with the long-term regulation of hypothalamic hormone on body weight, HGS can quickly and timely regulate metabolic homeostasis.

The particular structure of the hypothalamus determines the high sensitivity and accuracy of HGS, because the arcuate nucleus (ARC) located in the medial basal hypothalamus, which contains the most important glucose-sensing cells we have found so far, mainly POMC neurons and AGRP neurons (12), is very close to the median eminence without the blood-brain barrier (13). This structural basis makes it easier for passive diffusion of body fluid to transmit nutrient and energy signals to neural networks close to the ARC without being limited like other parts of the brain. Thus, ARC can sense changes in peripheral energy metabolism earlier and then transmit information to other regions of the hypothalamus and brain. The HIF complex plays an essential role in the glucose-dependent hypothalamic control of feeding and, energy balance and hypothalamic glucose sensing based on the above structures. We can note that the functional relationship between them is not unidirectional.

Glucose levels can affect HIF in the central nervous system. In the hypothalamus, HIF can be upregulated by glucose to achieve feeding regulation through glucose sensing. There are two pathways, including recruitment of AMPK and mTOR/S6K to regulate HIF-2α protein synthesis and inhibition of PHD to prevent HIF-2α degradation (3). Furthermore, we should know that not only glucose but also its metabolites can regulate HIF in the central nervous system. Pyruvate has been reported to inhibit PHD to stabilize HIF (14). In addition, succinate and fumarate were found to upregulate the protein level of HIF-2α through inhibition of PhD (10). Through these TCA cycle intermediates, increased HIF-2α is involved in hypothalamic glucose sensing.

On the other hand, many studies have shown that HIF can mediate the activity of GLUT, which is an essential glucose channel for mediating life activities. GLUT-1, GLUT-2, and GLUT-4 are all expressed in the brain (15). GLUT-1 mainly exists in microvascular endothelial cells and astrocytes (16), while GLUT-3 and GLUT-4 mainly exist in neurons (17) GLUT-1 is the main glucose transporter in the brain, responsible for promoting glucose transport through BBB (18). Mice that did not express GLUT-2 in the brain had glucagon imbalance, and the re-expression of GLUT-2 in their astrocytes restored the correct control of glucagon levels (19). GLUT is involved in the process of HGS: Based on the description of “neuron sensor” model, neurons absorb glucose through GLUT, and then glucose is metabolized into ATP, which is considered as a factor providing glucose concentration information. It binds and closes the ATP dependent potassium channel (KATP) widely expressed in POMC and AgRP neurons (20), leading to reduced potassium outflow and neuronal depolarization. HIF-1α is a transcription factor of GLUT-1, GLUT-3 (21), and an activator of GLUT-4 (22). Under hypobaric hypoxia, the expression of GLUT-1 was similar to that of HIF-1α (23–25), both of them were upregulated (26). No one has directly studied whether HIF can increase GLUT-2 in the brain, but in the liver, the increase of GLUT-2 is due to the up-regulation of HIF-1α (27), so the similar mechanism in the brain is also worth looking forward to. There is a persuasive evidence that HIF-1α regulates GLUT-4 in the brain: 2 month administration of α-lipoic acid (LA) can inhibit the development of Alzheimer’s disease, it can also significantly increase the protein and mRNA levels of GLUT-3, GLUT-4, vascular endothelial growth factor (VEGF) and heme oxygenase-1 (HO-1) in the brain of P301S mice (a tauopathy and AD mouse model) (28); LA induced activation of brain-derived neurotrophic factor (BDNF)/tyrosine kinase receptor B (TrkB)/HIF-1α signaling pathway may be one of the most important mechanisms during the above process (28).

In general, high glucose levels increase the expression of HIF in glucose sensing cells, which are also appetite control neurons. Increased HIF affects these neurons and reduces glucose intake. The glucose sensitivity of these neurons is at least partly based on the amount of GLUT that can be regulated by HIF. In astrocytes, HIF also increases GLUT and leads to more glucose absorption and sensing, which contributes to correct glucose regulation and reduces systemic glucose levels. In the whole-body glucose regulation process, it is not only the appetite control pathway that plays a role. In addition to adjusting food intake, HGS also participates in energy homeostasis by activating insulin secretion (IS) through vagal nerve. For example, high glucose levels in the hypothalamus stimulate IS and glycogen storage, while preventing hepatic glucose production (29).

## Hypoxia-Inducible Factors Induced Decrease of Hypothalamus NF-κB Relative Inflammation

Metabolic disorders are often accompanied by inflammation in CNS (30), which can be caused by infection, high-fat diet (HFD) and hypoxia, among which HFD is the most common cause. Therefore, diet-induced obesity (DIO) in rodent model induced by HFD is one of the most widely used models to study human obesity. In the pursuit of a better treating method for obesity, it is necessary to investigate the hypothalamic inflammatory process caused by HFD. In terms of inflammation, we must mention NF-κB because it is a key protein in inflammation and is always induced by HFD.

**Figure 1:**
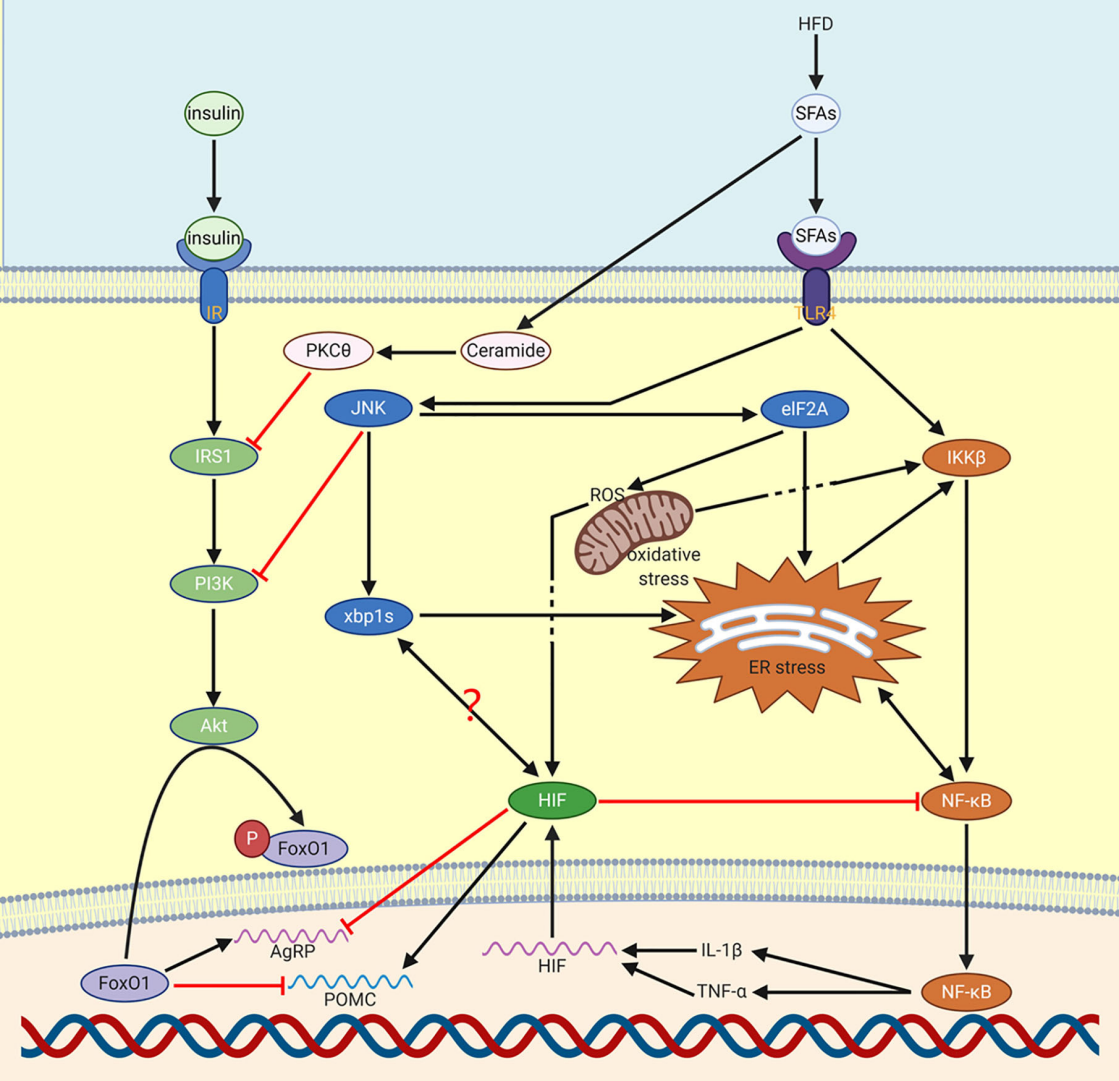
Production of NF-κB *via* HFD. HFD increases NF-κB through SFAs. Through JNK, ER stress can be activated. Meanwhile, NF-κB and ER stress will mediate each other. NF-κB induced IL-1β and TNF-α mediate the expression of HIF. Besides, SFAs induced PKCθ and JNK reduce the function of insulin in CNS. HIF activated POMC reduces food consumption in order to decrease the influence of HFD. On the contrary, AgRP will be inhibited by HIF. It’s possible that there is a relation between XBP1s and HIF.

The reason why HFD induces inflammation remains controversial. A well-known and compelling explanation is about long-chain saturated fatty acids (SFA), which have the same functional structure as lipopolysaccharide (LPS), which is responsible for binding to TLR4 (a pattern recognition receptor that recognizes molecular patterns associated with pathogens). For NF-κB, some studies have shown that SFAs activates glial proliferation of microglia and astrocytes to regulate inflammatory response (5), in this process, the LPS functional part that is composed of acylated SFA acts on TLR4 to activate NF-κB (**Figure 1**).

The activation of NF-κB induced by HFD can occur in microglia and neurons (5). For microglia, the activation of the NF-κB pathway leads to the recruitment of pro-inflammatory microglia in the hypothalamus and leads to obesity (5, 31, 32). For neurons, HFD causes neuronal stress, insulin sensitivity and leptin sensitivity reduction through the increase of NF-κB signal (33), which in turn weakens appetite inhibition and promotes overeating and intake of more HFD. For the precursor of neurons, the activation of IKKβ/NF-κB in hypothalamic neural stem cells (htNSCs) can prevent neuronal differentiation and induce consumptively damage of htNSCs, and eventually lead to the development of obesity and prediabetes (34). In addition, chronic HFD feeding impairs the function of htNSCs, HFD also activates apoptosis pathways in these cells triggered by IKKβ/NF-κB, which reduces the survival and proliferation of htNSCs (34). The injury of adult htNSCs mediated by IKKβ/NF-κB is an important neurodegenerative process in obesity and related diabetes mellitus (34).

There are at least two pathways *via* which NF-κB can activate HIF. One is an indirect way: in microglia whose main function is to communicate continuously with neurosecretory cells in the hypothalamus through IL-1β and TNF-α (pro-inflammatory cytokines), the activated IKKβ/NF-κB system can upregulate IL-1β and TNF-α, which can stabilize HIF activity by inhibiting PHD enzyme (4). Another is by NF-κB directly activating HIF: this method is not based on the effect on protein structure but is based on the mRNA expression of HIF, which can be promoted by the transport of NF-κB into the nucleus. On the opposite, HIF-1α cannot be effectively transcribed in infection or inflammation when NF-κB gene is deleted. Therefore, in the absence of NF-κB gene, HIF-1α will not have stability or activity even if exposed to adverse factors for a long time (6).

In model organisms Drosophila melanogaster and mammalian cells, the expression of NF-κB-dependent genes is inhibited by HIF-1α, such an inhibitory function is evolutionarily conserved (6). Direct deletion of HIF-1α can lead to increased NF-κB transcriptional activity by mechanisms depend on TAK and IKK as well as CDK6 (6). Inhibition of HIF-1α makes Drosophila more susceptible to death by inflammation after infection, suggesting that HIF-1α is involved in an important process of negative feedback inhibition on the NF-κB dependent defensive mechanism to achieve moderate immunity.

In the central nervous system, microglia are the main responders to LPS (35). For HIF, conditionally knocking out of isoform-specific pyruvate kinase M2 (PKM2) in cells demonstrates that the stabilization of HIF-1α can be achieved by PKM2, a key factor induced by LPS (36, 37). Therefore, LPS is a common upstream regulatory factor of HIF and NF-κB. During the induction by LPS, HIF is more likely to increase at the same time as NF-κB, rather than only after the increase of NF-κB as negative feedback. However, HIF is not the only factor that prevents NF-κB, because, in the absence of HIF, the termination of the NF-κB response still exists, which indicates that all these negative feedback points still exist (6).

Overall, in the hypothalamus, the key site of energy homeostasis regulation, central nervous system response to HFD and a high-sugar diet is primarily a quick upregulation of the NF-κB pathway. Deletion or inhibition of neuronal NF-κB pathway intermediates restores hypothalamic control of energy balance, thereby reducing the incidence of glucose intolerance and DIO (38). There are also many specific mechanisms, with which HIF prevents HFD from damaging the body, remain to be studied, some of which may be different from the crosstalk process we mentioned earlier. For example, in HFD fed mice, inhibition of HIF-1β in ARC resulted in significant weight gain and enhanced energy storage capacity (39).

## Hypoxia-Inducible Factors Induced Reactive oxygen species Production Decrease

Reactive oxygen species (ROS) are quite common substances in cells. The identified source of ROS is the mitochondrial respiratory chain complex. During aerobic respiration, oxygen is relocated to ROS after reacting with electrons. As a matter of fact, ROS can destroy human cells as well as bacteria. But the new role of ROS in diseases and health, especially metabolism, is attracting attention. In various peripheral organs, ROS are known to play a role in many signaling pathways. However, they also have a function in food intake regulation, metabolism and hormone secretion in the hypothalamus by affecting different types of neurons such as POMC and AgRP/NPY neurons (40). Different factors such as adipokines (leptin, apelin, etc.), pancreatic, intestinal hormones, nutrients (glucose, lipids, etc.) and neurotransmitters can affect the release of ROS in the hypothalamus. ROS released in CNS is involved in the development of many diseases, such as type 2 diabetes (T2D) (40).

In the brain, glucose, lipids and other common nutrients have a similar mechanism to induce mitochondrial ROS production in the hypothalamus alongside with an activity raise in mitochondrial respiration, so the central nervous system, after receiving lipid and glucose signals, can cause certain systemic metabolic regulation measures by upregulated ROS.

Also, lipid- Hypothalamic ROS can reduce food intake and increase insulin production. Through parasympathetic outflow, the release of ROS in the hypothalamus can induce an insulin peak after 1–3 min, without change in peripheral blood glucose (40). By using antioxidant molecules to clear ROS production in the brain, insulin secretion induced by brain glucose is significantly disturbed, which demonstrates the regulatory role of hypothalamic ROS in energy metabolism. ROS plays a specific role in different nerve cells. After glucose infused into ventromedial hypothalamus (VMH), through mROS production, the food intake is attenuated when re-feed after overnight fasting for a period (41). In ARC, ROS reduction mediates the activation of NPY/AgRP neurons, while ROS mediates the activation of POMC neurons (42). The ROS with the strongest effect on POMC neurons seems to be H_2_O_2_, increasing H_2_O_2_ can cause depolarization of POMC neurons, and ICV injection of H_2_O_2_ can cause significant anorexia. Furthermore, only ROS itself can mediate leptin action and restore POMC function (40).

## Reactive Oxygen Species Induced Increase of Hypoxia-Inducible Factor Stability

As one of the oxygen free radicals, ROS, can also regulate HIF. Low and moderate concentrations of ROS exert their functions, including regulation of HIF, by regulating cellular signaling cascades. It is worth noting that long-term inflammatory process can increase the production of ROS (43), so ROS may also play a role in DIO.

The HIF-α hydroxylation rate partly depends on the level of PhD (44). In the presence of oxygen, Fe2+, 2-oxoglutarate (2-OG) and ascorbic acid (45, 46), PhD enzymes are active and can hydroxylate conserved proline residues of the HIF-α subunit using alpha-ketoglutarate and molecular oxygen as co-substrates, which then lead to HIF-α’s proteasomal degradation under non-hypoxic conditions (47, 48). A quick increase in ROS (49)can be observed within the first minute of hypoxia, which helps stabilize HIF-α protein, mainly by oxidizing central Fe(II) to Fe(III) to promote PHD enzyme inactivation (50).

The stabilizing effect of ROS on HIFs is common, H_2_O_2_ is the most effective ROS to inhibit PHD activity and thus interfere with HIFs degradation (51), while other types of ROS from diverse complexes of mitochondria can stabilize HIF: knockdown of GRIM-19 (NDUFA13), a subunit of complex I, can induce ROS and then stabilize HIF-1α (50). Loss of SDHB, a subunit of the iron-sulfur cluster of complex II, causes HIF stabilization through a ROS-dependent pathway. Furthermore, only SDHB loss can trigger ROS formation and can stabilize HIF in mitochondrial complex II (52). In addition, the ROS of mitochondrial complex III can also stabilize HIF, but it is not clear whether complex III ROS is necessary to induce HIF (53–55). In complex III, RISP knockdown reduced HIF-1α expression and demonstrated a link between HIF and ROS of mitochondrial complex III (56).

There is evidence that HIF can increase the glycolytic rate by upregulating the transcription of glycolytic genes (1), which reduces oxygen consumption and reduces hypoxia-induced stress, then reduces inflammation that can increase ROS. In addition, other studies have shown that mammalian HIF-1α is necessary to control the production of toxic ROS in hypoxia, as HIF-1α can activate pyruvate dehydrogenase kinase 1 (6, 57), a key factor that decreases ROS. But there is another more specific regulatory process: REDD1 protein is an essential HIF-1α effector for regulating activity of mTOR complex 1 (mTORC1) in Drosophila and mammalian cells (58); REDD1 is also a key factor in controlling ROS production under hypoxia (6)—when localizes to the mitochondria, ROS production will be reduced by REDD1 (58). Intuitively, HIF reduces ROS production, but it cannot be considered that HIF and ROS are simply antagonistic to each other. Considering that some functions of HIF and ROS overlap, such as H_2_O_2_, which has the strongest effect on POMC neurons, is also the most effective ROS to interfere with the degradation of HIF, this strongly indicates that HIF is more suitable to be identified as a downstream factor regulated by ROS, plays a part of the regulatory role caused by ROS, and exerts negative feedback regulation on ROS.

## Hypoxia-Inducible Factors Induced Increase of Leptin Sensitivity

Leptin is produced by peripheral adipose tissue and plays a role in the CNS, which cannot be ignored when investigating the development of metabolic diseases. Leptin signals activate Janus-activated kinase (JAK)-2 through LEPR and promote later signaling through effector cascades, including signal transduction and activators in the phosphatidylinositol 3-kinase (PI3K)/AKT and transcription activator (STAT)-3 pathways, to increase POMC expression and inhibit AGRP expression, thereby promoting satiety to suppress food intake and promote energy expenditure (59). In addition, POMC promotes white fat browning, which can be caused by simultaneous activation of leptin and insulin signaling pathways in POMC neurons (60). Through the activation of POMC, leptin can also increase the secretion of α-MSH, thereby directly activating MC4R in the paraventricular hypothalamic nucleus (PVH), dorsal medial thalamus(DMH) and intermediolateral nucleus (IML), which can mediate the peripheral sympathetic outflow of SNA through direct and indirect signaling processes, ultimately increasing mitochondrial UCP-1 expression and BAT activity, resulting in increased heat production in BAT, which is one of the mechanisms that leptin increases energy expenditure.

Overall, HIF can regulate leptin and insulin simultaneously through several pathways. There are also some independent regulatory pathways for common pathways that regulate both insulin and leptin.

The relationship between HIF and leptin in the central nervous system is less clear than the one between HIF and insulin. According to existing research conclusions, HIF regulates leptin through suppressor of leptin signaling (SOCS), one of the hypothalamic signaling molecules/pathways that have been extensively studied to inhibit obesity-induced leptin resistance (61). This process involves several elements. Activated NF-κB can cause an increase in the expression of SOCS3, which prevents insulin (33) and leptin (62) signaling in the brain. This process is obvious and rapid, once exposed to HFD, overexpression of SOCS3 can be detected in AgRP neurons, inducing energy imbalance, leptin, and insulin resistance, and hyper-appetite (63). According to our conclusion, if HIF reduces NF-κB, then SOCS3 will also be reduced by HIF, then leptin and insulin sensitivity will increase. In addition, HIF can increase leptin and insulin sensitivity by reducing ER stress, which conversely regulates HIF through the PI3K/AKT pathway, as we will describe in the insulin section later.

## Hypoxia-Inducible Factors Induced Increase of Insulin Sensitivity

Insulin is one of the most critical hormones in the development of metabolic diseases. Insulin affects systemic metabolism and has special effects on the hypothalamus. In the peripheral body part, when blood glucose rises, insulin is released from pancreatic β cells and enters the central nervous system through the blood-brain barrier. In the central nervous system, insulin receptors can be found throughout the brain, especially in neurons of the hypothalamus, where insulin mainly acts on POMC and AgRP/NPY neurons of ARC. Through ligand-induced stimulation, insulin upregulates POMC expression and reduces food intake (4). Knockdown of insulin receptors in the central nervous system leads to gender-specific mild obesity, and obese males are resistant to insulin-mediated anorexia function imply both peripheral and central insulin resistance are included in the development of obesity (20).

HIF is one of the downstream pathways of insulin function. Indeed, both insulin and leptin can regulate HIF through the common PI3K/AKT pathway. Here, we will discuss the detailed process.

**Figure 2:**
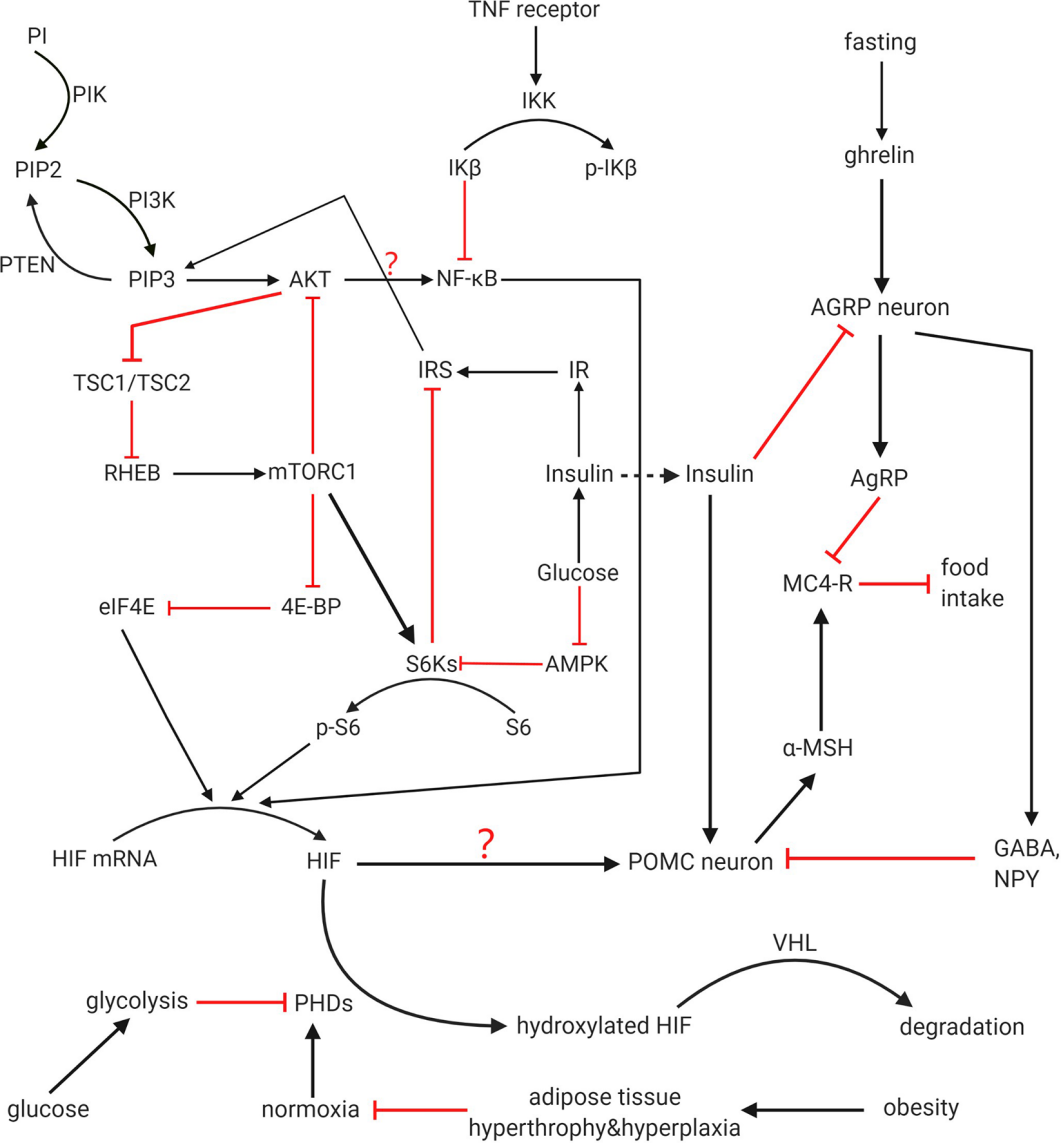
HIF regulation and function on POMC. PI3K pathway, HIF-PHD-VHL pathway and mTOR/NF-kB/HIF pathway all participates in regulation of HIF. Crosstalk among these pathways is not completely clear. PI3K, mTOR, and NF-kB can increase expression of HIF, while PHD negatively regulate HIF and promote its degradation with VHL as a cofactor. Hypoxia among adipocytes caused by obesity downregulated the activity of PHDs. HIF works as one of the activators of POMC gene that prevent food intake. Whether the feedback from IRS to PIP3 and the function from AKT to NF-kB can finally influence the activity of HIF is not clear and demand further study. Whether these upstream activated HIF can activate all the functional effects of POMC has not been directly stated in previous articles, and it is for further study.

PI3K/AKT/mTOR signaling pathway is one of the main upstream regulatory pathways of HIF-1α. Through this pathway, not only the synthesis of new HIF-1α protein is increased due to the increase of translation, but also Hsp90, which maintains the stability of HIF-1α protein to prevent its degradation, is also activated and enhanced (64). The pathway itself has negative feedback, activated AKT can increase the expression of mTOR, and chronic activation of mTOR complex 1 (mTORC1) signal transduction can inhibit IRS-1, then Akt signal transduction will be reduced (65) (**Figure 2**).

**Figure 3:**
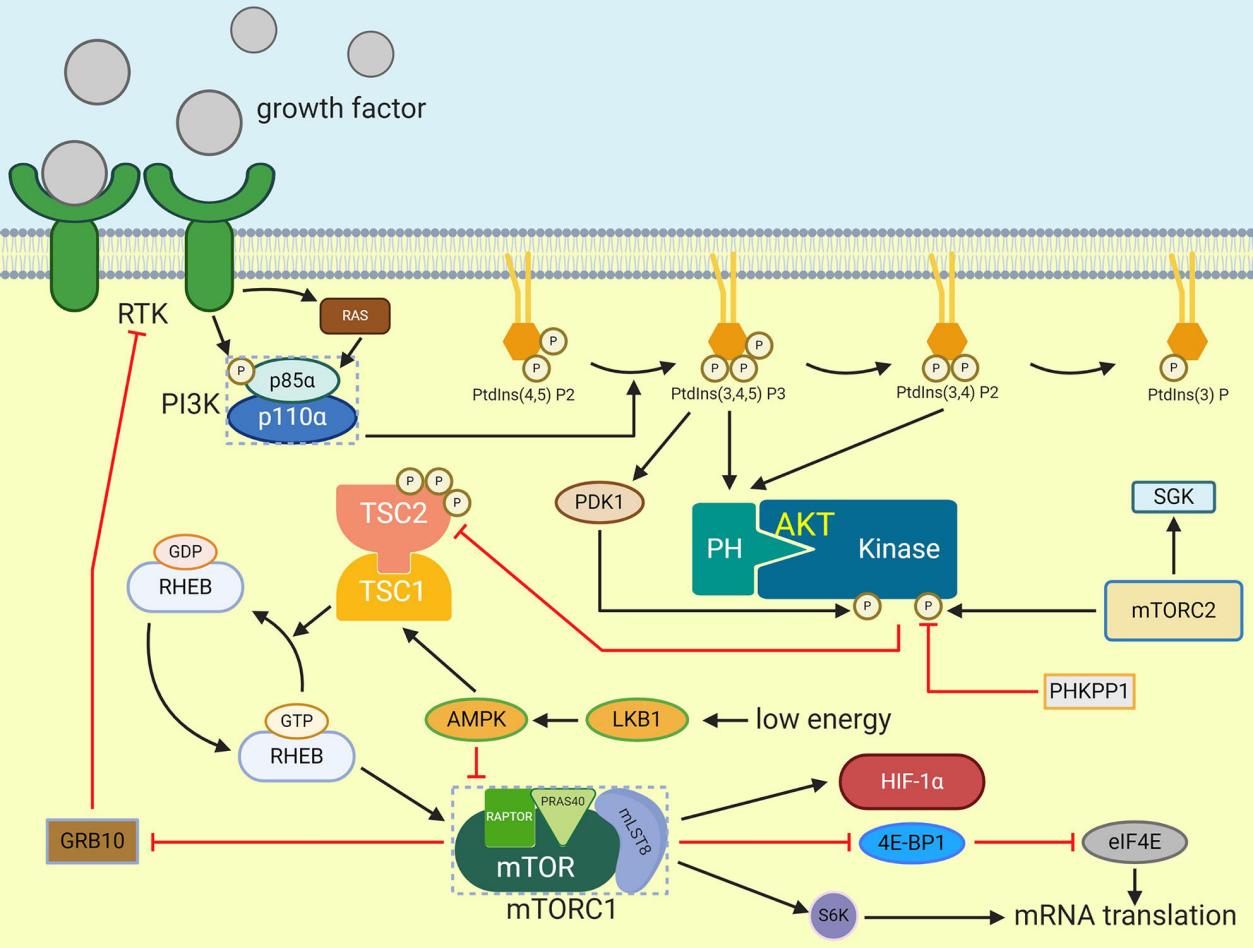
Activation of mTORC1. Extracellular signals such as growth factor can stimulate receptor that activate PI3K which will cause PIP3 generation. PIP3 then activate AKT by phosphorylation. TSC2 is phosphorylated by AKT and then inactivate TSC1/TSC2 complex which prevent RHEB activation. After that, GTP-bounded RHEB will activate mTORC1. eIF4E-binding protein 1 and S6K can be phosphorylated by activated mTORC1, and they can enhance mRNA translation. HIF-1α is one of the mTORC1 effectors.

MTOR can be considered as an important part of energy homeostasis regulated by CNS because it is a downstream target of PI3K pathway stimulated by insulin and has the function of regulating glucose/lipid homeostasis, body weight and energy consumption through hypothalamus (66). These functions are embodied in the hypothalamus mTORC1 through reducing the expression of AgRP and NPY, thereby reducing food intake and body weight. On the contrary, over nutrition down-regulates the activity of mTORC1 in the hypothalamus, and then causes leptin resistance, weight gain and excessive appetite. According to the existing studies, there is a significant relationship between mTOR and HIF. mTOR can directly improve the translation rate of HIF-1α mRNA through 5’oligopyrimidine nucleotide sequence, thus promoting the expression of HIF-1α. MTOR/S6K can promote HIF-2α protein synthesis, as the increased S6K activity will lead to the up-regulation of HIF-2α in hypothalamus when glucose is provided (3). In addition, mTORC1 can phosphorylate the binding protein 1 (4E-BP1) of eukaryotic initiation factor 4E (eIF4E) to terminate the inhibition of the latter by the former (67), then the expression of HIF will be promoted by the release of eIF4E (**Figure 3**).

HIF does assist in insulin’s activity in multiple ways. In addition to inhibiting SOCS3, there are at least two methods. After stimulating the CNS with insulin, the level of HIF-2α protein in the hypothalamus of young mice increased rapidly compared with aged mice, while the activation of IR/IRS-2/Akt/FoxO1 pathway in aged mice and young mice was similar (10). This indicates the mechanism that age decreases the level of upregulating HIF-2α mediated by insulin may adopt another pathway different from IR/IRS-2/Akt/FoxO1. Like the ageing mice, HIF-2α is not increasing that much in short-term DIO mice (10). According to the analysis of physiology and molecular structure, HIF-2α can maintain the function of central insulin and promote the expression of the POMC gene. These results suggest that HIF-2α knockout in POMC neurons can cause insulin resistance and glucose intolerance, and lead to age-dependent weight gain and fat gain.

Secondly, HIF can increase insulin and leptin sensitivity by reducing endoplasmic reticulum (ER) stress. Unfolded protein response (UPR) caused by ER dysfunction is known as ER stress, which is related to metabolic diseases including insulin resistance and obesity (5) because induced ER stress prevents weight loss and anorexic functions of insulin and leptin (68). Considering that UPR may promote HIF through inositol-requiring enzyme 1α (IRE1α)-X box-binding protein 1 (XBP1) (69, 70), while IKK/NF-κB and ER stress promote each other and induce energy imbalance leading to obesity during HFD feeding (68), it can be inferred that HIF can act as downstream negative feedback to inhibit NF-κB in the process of ER stress, so as to reduce the adverse effects of this stress.

## Increasing of Spontaneous Physical Activity by Orexin *via* Induced Increase of Insulin Sensitivity

The hypothalamic neuropeptides orexin-A and orexin-B are the cleavage products of prepro-orexin (PPO). The immunoreactive neurons to OXA and OXB antibodies were mainly located in the dorsomedial (DMH), lateral (LH) and perifornical hypothalamus (PeF) of rat, human and pig (71–73). In situ hybridization data showed that OXRs (orexin receptor-1 and -2) were widely located in the brainstem, cerebral cortex, hypothalamus and thalamus (74, 75).

There is an obvious relationship between orexin-A, orexin B and hypothalamic neuropeptides, as orexin responds to metabolic, limbic, and circadian stimuli by activating cholinergic and monoaminergic neurons in the brainstem and hypothalamus to maintain wakefulness or sleep or alertness (76), sense nutrients and regulate appetite (77). Via these processes, orexin enables the living body to achieve various changes that should be made to adapt to the environment and regulate the organism itself.

Among this series of adaptive and regulatory roles, another review highlights the ability of orexin-A to take part in counteracting the brain mechanisms responsible for obesity (78). Spontaneous physical activity (SPA) is positively correlated with obesity resistance, during SPA, the increase of non-exercise activity thermogenesis (NEAT) is the main mechanism (79). Second, there is evidence of orexin ability to increase NEAT (78). By combining these two facts, the potential of orexin in combating obesity cannot be denied. According to the above viewpoints, orexin is of great concern to researchers who want to study the mechanisms involved in combating obesity.

For a long time, many experiments have found that HIF is related to cancer and hypoxia. However, OXA has recently been proved to increase HIF-1α activity by downregulating VHL and increasing the transcription of HIF-1α gene to induce the expression of HIF-1α (77, 78). The increase of HIF-1α can also be achieved by OXA in a more indirect but specific way: OXA has been proved to activate mitogen-activated protein kinase (MAPK) pathway. Higher MAPK activity can make mice express higher levels of peroxisome proliferator-activated receptor-gamma coactivator 1 alpha (PGC-1α) in skeletal muscle. The latter is expressed throughout the brain, and its changes are associated with obesity, diabetes mellitus, pathological changes such as chronic neurodegenerative diseases (78). PGC-1α is involved in regulating HIF-1α in peripheral tissues (80), and it is speculated that the same process can be done in the hypothalamus (78). Orexin mediated HIF-1α activation leads to higher glycolysis activity and increased glucose uptake; it is an important transcription factor in mediating hormone-induced arousal and starvation (77). Data from several independent *in vitro* and rodent models support the hypothesis that OXA mediated increase of energy consumption suggests orexin’s obesity resistance may be partially dependent on signaling cascades involving MAPK, PGC-1α, and HIF-1α, regulation of these pathways ultimately induces increased SPA and obesity resistance (78). In addition, anoxia induced orexin function decreasing was identified as one of the functions of HIF. Orexin reduces the expression of lactate dehydrogenase A (LDHA) and pyruvate dehydrogenase kinase 1 (PDK1) genes (LDHA converted pyruvate to lactic acid, while PDK1 phosphorylated PDH, both of which promoted pyruvate flow through anaerobic glycolysis), and when HIF-1α was knocked down, ldhA and PDK1 gene expression were even lower (77).

## Conclusion

HIF plays an important role in hypothalamus regulation and affects systemic metabolism. Nutrition sensing, hormone induction, metabolic regulation and appetite regulation are important functions of HIF that have been discovered.

Neural cells based mainly in ARC control appetite and energy expenditure, loss of HIF will at least partially disturb these controls. However, it is not sure if appetite and energy expenditure are modulated by neurons *via* the same mechanism; after all, the target organs are different. The importance of HIF in AGRP neurons has not been studied. In addition, glucose is demonstrated to influence the stability of HIF protein, but the relation between central HIF and other nutrients like lipids and amino acids is unclear. Hence, future studies are still necessary to answer these questions.

Although most of our understanding of the physiological principles and therapeutic potential of the HIF pathways comes from laboratory studies, information obtained from these non-clinical trials will provide *in vivo* pharmacological information on agents that mimic or regulate the function of HIF. From these known contents, we can also infer that HIF has the potential to become a focus in metabolic research. This is an exciting period based on our rapidly evolving understanding of the central nervous system’s role in regulating metabolism and energy balance throughout the body. Given the evidence that HIF plays an essential role in the central regulation of metabolism, the multiple effects of related drugs on metabolic diseases are likely to become an important clinical research field soon. More pharmacological, biological, and clinical experiments should be carried out to study the details of molecular signal transduction and the mechanisms involved in changing cellular responses caused by HIF in the hypothalamus and any other relevant parts of the CNS. The relation between these factors is not fully clear. A better understanding of the upstream factors that regulate hypothalamic activity can provide more options for preventing obesity and many other related metabolic diseases in the future.

## Author Contributions

All authors have debated the proposed scope and content of the article before drafting. All authors contributed to the article and approved the submitted version.

## Funding

This study was supported by the National Natural Science Foundation of China (81974117) and (81774035); Guangdong Basic and Applied Basic Research Foundation (Grant No. 2019A1515010665).

## Conflict of Interest

The authors declare that the research was conducted in the absence of any commercial or financial relationships that could be construed as a potential conflict of interest.

## References

[1] Taylor CT Colgan SP. Regulation of immunity and inflammation by hypoxia in immunological niches. Nat Rev Immunol (2017) 17(12):774–85. 10.1038/nri.2017.103 PMC5799081 28972206

[2] Xie C Yagai T Luo Y Liang X Chen T Wang Q. Activation of intestinal hypoxia-inducible factor 2α during obesity contributes to hepatic steatosis. Nat Med (2017) 23(11):1298–308. 10.1038/nm.4412 PMC6410352 29035368

[3] Zhang H Zhang G Gonzalez FJ Park SM Cai D. Hypoxia-Inducible Factor Directs POMC Gene to Mediate Hypothalamic Glucose Sensing and Energy Balance Regulation. PLoS Biol (2011) 9(7)e1001112. 10.1371/journal.pbio.1001112 PMC3144184 21814490

[4] Gaspar JM Velloso LA. Hypoxia Inducible Factor as a Central Regulator of Metabolism – Implications for the Development of Obesity. Front Neurosci Switz (2018) 12:813. 10.3389/fnins.2018.00813 PMC6221908 30443205

[5] Seong J Kang JY Sun JS Kim KW. Hypothalamic inflammation and obesity: a mechanistic review. Arch Pharm Res (2019) 42(5):383–92. 10.1007/s12272-019-01138-9 30835074

[6] Bandarra D Biddlestone J Mudie S Müller HAJ Rocha S. HIF-1α restricts NF-κB-dependent gene expression to control innate immunity signals. Dis Model Mech (2015) 8(2):169–81. 10.1242/dmm.017285 PMC4314782 25510503

[7] Minamishima YA Moslehi J Padera RF Bronson RT Liao R Kaelin WJ. A feedback loop involving the Phd3 prolyl hydroxylase tunes the mammalian hypoxic response in vivo. Mol Cell Biol (2009) 29(21):5729–41. 10.1128/MCB.00331-09 PMC2772748 19720742

[8] Briggs KJ Koivunen P Cao S Backus KM Olenchock BA Patel H. Paracrine Induction of HIF by Glutamate in Breast Cancer: EglN1 Senses Cysteine. Cell (2016) 166(1):126–39. 10.1016/j.cell.2016.05.042 PMC4930557 27368101

[9] Shukla SK Purohit V Mehla K Gunda V Chaika NV Vernucci E. MUC1 and HIF-1alpha Signaling Crosstalk Induces Anabolic Glucose Metabolism to Impart Gemcitabine Resistance to Pancreatic Cancer. Cancer Cell (2017) 32(1):71–87. 10.1016/j.ccell.2017.06.004 28697344 PMC5533091

[10] Wang Z Khor S Cai D. Age-dependent decline of hypothalamic HIF2α in response to insulin and its contribution to advanced age-associated metabolic disorders in mice. J Biol Chem (2019) 294(13):4946–55. 10.1074/jbc.RA118.005429 PMC6442045 30709906

[11] López-Gambero AJ Martínez F Salazar K Cifuentes M Nualart F. Brain Glucose-Sensing Mechanism and Energy Homeostasis. Mol Neurobiol (2019) 56(2):769–96. 10.1007/s12035-018-1099-4 29796992

[12] Abizaid A Horvath TL. Brain circuits regulating energy homeostasis. Regul Pept (2008) 149(1–3):3–10. 10.1016/j.regpep.2007.10.006 18514925 PMC2605273

[13] Kalra SP Dube MG Pu S Xu B Horvath TL Kalra PS. Interacting appetite-regulating pathways in the hypothalamic regulation of body weight. Endocr Rev (1999) 20(1):68–100. 10.1210/edrv.20.1.0357 10047974

[14] Virtue S Vidal-Puig A. Nothing Iffy about HIF in the Hypothalamus. PloS Biol (2011) 9(7):e1001116. 10.1371/journal.pbio.1001116 21814494 PMC3144187

[15] Mueckler M Thorens B. The SLC2 (GLUT) family of membrane transporters. Mol Aspects Med (2013) 34(2-3):121–38. 10.1016/j.mam.2012.07.001 PMC4104978 23506862

[16] Simpson IA Appel NM Hokari M Oki J Holman GD Maher F. Blood-brain barrier glucose transporter: effects of hypo- and hyperglycemia revisited. J Neurochem (1999) 72(1):238–47. 10.1046/j.1471-4159.1999.0720238.x 9886075

[17] Simpson FMSJ. Glucose transporter proteins in brain. FASEB J (1994) 8 (13):1003–11. 10.1096/fasebj.8.13.7926364 7926364

[18] García-Cáceres C Quarta C Varela L Gao Y Gruber T Legutko B. Astrocytic Insulin Signaling Couples Brain Glucose Uptake with Nutrient Availability. Cell (2016) 166(4):867–80. 10.1016/j.cell.2016.07.028 PMC8961449 27518562

[19] Marty N. Regulation of glucagon secretion by glucose transporter type 2 (glut2) and astrocyte-dependent glucose sensors. J Clin Invest (2005) 115(12):3545–53. 10.1172/JCI26309 PMC1297256 16322792

[20] Belgardt BF Okamura T Brüning JC. Hormone and glucose signalling in POMC and AgRP neurons. J Physiol (2009) 587(22):5305–14. 10.1113/jphysiol.2009.179192 PMC2793863 19770186

[21] Denko NC. Hypoxia, HIF1 and glucose metabolism in the solid tumour. Nat Rev Cancer (2008) 8(9):705–13. 10.1038/nrc2468 19143055

[22] Sakagami H Makino Y Mizumoto K Isoe T Takeda Y Watanabe J. Loss of HIF-1α impairs GLUT4 translocation and glucose uptake by the skeletal muscle cells. Am J Physiol: Endocrinol Metab (2014) 306(9):E1065–76. 10.1152/ajpendo.00597.2012 24619881

[23] Harik N Harik SI Kuo NT Sakai K Przybylski RJ LaManna JC. Time-course and reversibility of the hypoxia-induced alterations in cerebral vascularity and cerebral capillary glucose transporter density. Brain Res (1996) 737(1-2):335–8. 10.1016/0006-8993(96)00965-1 8930387

[24] Kuo NT Benhayon D Przybylski RJ Martin RJ LaManna JC. Prolonged hypoxia increases vascular endothelial growth factor mRNA and protein in adult mouse brain. J Appl Physiol (1985) (1999) 86(1):260–4. 10.1152/jappl.1999.86.1.260 9887138

[25] Xu F Severinghaus JW. Rat brain VEGF expression in alveolar hypoxia: possible role in high-altitude cerebral edema. J Appl Physiol (1985) (1998) 85(1):53–7. 10.1152/jappl.1998.85.1.53 9655755

[26] Chavez JC Agani F Pichiule P LaManna JC. Expression of hypoxia-inducible factor-1alpha in the brain of rats during chronic hypoxia. J Appl Physiol (1985) (2000) 89(5):1937–42. 10.1152/jappl.2000.89.5.1937 11053346

[27] Sacramento JF Ribeiro MJ Rodrigues T Guarino MP Diogo LN Seiça R. Insulin resistance is associated with tissue-specific regulation of HIF-1α and HIF-2α during mild chronic intermittent hypoxia. Respir Physiol Neurobiol (2016) 228:30–8. 10.1016/j.resp.2016.03.007 26993367

[28] Zhang Y Yan X Xu S Pang Z Li L Yang Y. α-Lipoic Acid Maintains Brain Glucose Metabolism via BDNF/TrkB/HIF-1α Signaling Pathway in P301S Mice. Front Aging Neurosci (2020) 12:262. 10.3389/fnagi.2020.00262 32973490 PMC7471806

[29] Desmoulins L Chrétien C Paccoud R Collins S Cruciani-Guglielmacci C Galinier A. Mitochondrial Dynamin-Related Protein 1 (DRP1) translocation in response to cerebral glucose is impaired in a rat model of early alteration in hypothalamic glucose sensing. Mol Metab (2019) 20:166–77. 10.1016/j.molmet.2018.11.007 PMC6358535 30553770

[30] Chen Y Yu X Liu K Gao H Li Y Sun T. Inhibition of Hypothalamic Inhibitor κB Kinase β/Nuclear Transcription Factor κB Pathway Attenuates Metabolism and Cardiac Dysfunction in Type 2 Diabetic Rats. Neuroendocrinology (2020) 110(11-12):899–913. 10.1159/000504444 31671427

[31] Andre C Guzman-Quevedo O Rey C Remus-Borel J Clark S Castellanos-Jankiewicz A. Inhibiting Microglia Expansion Prevents Diet-Induced Hypothalamic and Peripheral Inflammation. Diabetes (2017) 66(4):908–19. 10.2337/db16-0586 27903745

[32] Valdearcos M Douglass JD Robblee MM Dorfman MD Stifler DR Bennett ML. Microglial Inflammatory Signaling Orchestrates the Hypothalamic Immune Response to Dietary Excess and Mediates Obesity Susceptibility. Cell Metab (2017) 26(1):185–97. 10.1016/j.cmet.2017.05.015 PMC5569901 28683286

[33] Zhang X Zhang G Zhang H Karin M Bai H Cai D. Hypothalamic IKKβ/NF-κB and ER Stress Link Overnutrition to Energy Imbalance and Obesity. Cell (2008) 135(1):61–73. 10.1016/j.cell.2008.07.043 18854155 PMC2586330

[34] Li J Tang Y Cai D. IKKβ/NF-κB disrupts adult hypothalamic neural stem cells to mediate a neurodegenerative mechanism of dietary obesity and pre-diabetes. Nat Cell Biol (2012) 14(10):999–1012. 10.1038/ncb2562 22940906 PMC3463771

[35] Kumar H Lim J Kim I Choi D. Differential regulation of HIF-3α in LPS-induced BV-2 microglial cells: Comparison and characterization with HIF-1α. Brain Res (2015) 1610:33–41. 10.1016/j.brainres.2015.03.046 25847716

[36] Palsson-McDermott EM Curtis AM Goel G Lauterbach MAR Sheedy FJ Gleeson LE. Pyruvate Kinase M2 Regulates Hif-1α Activity and IL-1β Induction and Is a Critical Determinant of the Warburg Effect in LPS-Activated Macrophages. Cell Metab (2015) 21(1):65–80. 10.1016/j.cmet.2014.12.005 25565206 PMC5198835

[37] Luo W Hu H Chang R Zhong J Knabel M O’Meally R. Pyruvate kinase M2 is a PHD3-stimulated coactivator for hypoxia-inducible factor 1. Cell (2011) 145(5):732–44. 10.1016/j.cell.2011.03.054 PMC3130564 21620138

[38] Douglass JD Dorfman MD Fasnacht R Shaffer LD Thaler JP. Astrocyte IKKβ/NF-κB signaling is required for diet-induced obesity and hypothalamic inflammation. Mol Metab (2017) 6(4):366–73. 10.1016/j.molmet.2017.01.010 PMC5369266 28377875

[39] Gaspar JM Mendes NF Corrêa-da-Silva F Lima-Junior JCD Gaspar RC Ropelle ER. Downregulation of HIF complex in the hypothalamus exacerbates diet-induced obesity. Brain Behav Immun (2018) 73:550–61. 10.1016/j.bbi.2018.06.020 29935943

[40] Drougard A Fournel A Valet P Knauf C. Impact of hypothalamic reactive oxygen species in the regulation of energy metabolism and food intake. Front Neurosci (2015) 9:56. 10.3389/fnins.2015.00056 25759638 PMC4338676

[41] Carneiro L Allard C Guissard C Fioramonti X Tourrel-Cuzin C Bailbé D. Importance of mitochondrial dynamin-related protein 1 in hypothalamic glucose sensitivity in rats. Antioxid Redox Signal (2012) 17(3):433–44. 10.1089/ars.2011.4254 22229526

[42] Andrews ZB Liu Z Walllingford N Erion DM Borok E Friedman JM. UCP2 mediates ghrelin’s action on NPY/AgRP neurons by lowering free radicals. Nat 2008 (7206) 454:846–51. 10.1038/nature07181 PMC4101536 18668043

[43] Harrison DG Bernstein KE. 7 - Inflammation and Immunity in Hypertension. In: Bakris GL Sorrentino MJ, editors. Hypertension: A Companion to Braunwald"s Heart Disease, 3rd ed. Elsevier (2018). p. 60–69.

[44] Di Conza G Trusso CS Loroch S Mennerich D Deschoemaeker S Di Matteo M. The mTOR and PP2A Pathways Regulate PHD2 Phosphorylation to Fine-Tune HIF1α Levels and Colorectal Cancer Cell Survival under Hypoxia. Cell Rep (2017) 18(7):1699–712. 10.1016/j.celrep.2017.01.051 PMC5318657 28199842

[45] Zhao L Liu Z Yang F Zhang Y Xue Y Miao H. Intrabody against prolyl hydroxylase 2 promotes angiogenesis by stabilizing hypoxia-inducible factor-1α. Sci Rep UK (2019) 9(1):11861. 10.1038/s41598-019-47891-1 PMC6694103 31413262

[46] Koivunen P Serpi R Dimova EY. Hypoxia-inducible factor prolyl 4-hydroxylase inhibition in cardiometabolic diseases. Pharmacol Res (2016) 114:265–73. 10.1016/j.phrs.2016.11.003 27832958

[47] Rey S Semenza GL. Hypoxia-inducible factor-1-dependent mechanisms of vascularization and vascular remodelling. Cardiovasc Res (2010) 86(2):236–42. 10.1093/cvr/cvq045 PMC2856192 20164116

[48] Kaelin WJ Ratcliffe PJ. Oxygen sensing by metazoans: the central role of the HIF hydroxylase pathway. Mol Cell (2008) 30(4):393–402. 10.1016/j.molcel.2008.04.009 18498744

[49] Hernansanz-Agustín P Izquierdo-Álvarez A Sánchez-Gómez FJ Ramos E Villa-Piña T Lamas S. Acute hypoxia produces a superoxide burst in cells. Free Radical Bio Med (2014) 71:146–56. 10.1016/j.freeradbiomed.2014.03.011 24637263

[50] Fuhrmann DC Brüne B. Mitochondrial composition and function under the control of hypoxia. Redox Biol (2017) 12:208–15. 10.1016/j.redox.2017.02.012 PMC5333533 28259101

[51] Dehne N Brüne B. Sensors, transmitters, and targets in mitochondrial oxygen shortage-a hypoxia-inducible factor relay story. Antioxid Redox Signal (2014) 20(2):339–52. 10.1089/ars.2012.4776 22794181

[52] Guzy RD Sharma B Bell E Chandel NS Schumacker PT. Loss of the SdhB, but Not the SdhA, Subunit of Complex II Triggers Reactive Oxygen Species-Dependent Hypoxia-Inducible Factor Activation and Tumorigenesis. Mol Cell Biol (2008) 28(2):718–31. 10.1128/MCB.01338-07 PMC2223429 17967865

[53] Comito G Calvani M Giannoni E Bianchini F Calorini L Torre E. HIF-1α stabilization by mitochondrial ROS promotes Met-dependent invasive growth and vasculogenic mimicry in melanoma cells. Free Radic Biol Med (2011) 51(4):893–904. 10.1016/j.freeradbiomed.2011.05.042 21703345

[54] Klimova T Chandel NS. Mitochondrial complex III regulates hypoxic activation of HIF. Cell Death Differ (2008) 15(4):660–6. 10.1038/sj.cdd.4402307 18219320

[55] Chua YL Dufour E Dassa EP Rustin P Jacobs HT Taylor CT. Stabilization of Hypoxia-inducible Factor-1α Protein in Hypoxia Occurs Independently of Mitochondrial Reactive Oxygen Species Production. J Biol Chem (2010) 285(41):31277–84. 10.1074/jbc.M110.158485 PMC2951202 20675386

[56] Brunelle JK Bell EL Quesada NM Vercauteren K Tiranti V Zeviani M. Oxygen sensing requires mitochondrial ROS but not oxidative phosphorylation. Cell Metab (2005) 1(6):409–14. 10.1016/j.cmet.2005.05.002 16054090

[57] Kim J Tchernyshyov I Semenza GL Dang CV. HIF-1-mediated expression of pyruvate dehydrogenase kinase: A metabolic switch required for cellular adaptation to hypoxia. Cell Metab (2006) 3(3):177–85. 10.1016/j.cmet.2006.02.002 16517405

[58] Horak P Crawford AR Vadysirisack DD Nash ZM DeYoung MP Sgroi D. Negative feedback control of HIF-1 through REDD1-regulated ROS suppresses tumorigenesis. Proc Natl Acad Sci USA (2010) 107(10):4675–80. 10.1073/pnas.0907705107 PMC2842042 20176937

[59] Myers MG Cowley MA Münzberg H. Mechanisms of leptin action and leptin resistance. Annu Rev Physiol (2008) 70:537–56. 10.1146/annurev.physiol.70.113006.100707 17937601

[60] Dodd GT Decherf S Loh K Simonds SE Wiede F Balland E. Leptin and Insulin Act on POMC Neurons to Promote the Browning of White Fat. Cell (2015) 160(1-2):88–104. 10.1016/j.cell.2014.12.022 25594176 PMC4453004

[61] Kwon O Kim KW Kim MS. Leptin signalling pathways in hypothalamic neurons. Cell Mol Life Sci (2016) 73(7):1457–77. 10.1007/s00018-016-2133-1 PMC11108307 26786898

[62] Pedroso J Ramos-Lobo AM Donato JJ. SOCS3 as a future target to treat metabolic disorders. Hormones (Athens) (2019) 18(2):127–36. 10.1007/s42000-018-0078-5 30414080

[63] Olofsson LE Unger EK Cheung CC Xu AW. Modulation of AgRP-neuronal function by SOCS3 as an initiating event in diet-induced hypothalamic leptin resistance. Proc Natl Acad Sci (2013) 110(8):E697–706. 10.1073/pnas.1218284110 PMC3581908 23386726

[64] Choi YK Kim C Lee H Jeoung D Ha K Kwon Y. Carbon Monoxide Promotes VEGF Expression by Increasing HIF-1α Protein Level via Two Distinct Mechanisms, Translational Activation and Stabilization of HIF-1α Protein. J Biol Chem (2010) 285(42):32116–25. 10.1074/jbc.M110.131284 PMC2952213 20724477

[65] Albert V Hall MN. mTOR signaling in cellular and organismal energetics. Curr Opin Cell Biol (2015) 33:55–66. 10.1016/j.ceb.2014.12.001 25554914

[66] Hu F Xu Y Liu F. Hypothalamic roles of mTOR complex I: integration of nutrient and hormone signals to regulate energy homeostasis. Am J Physiol Endocrinol Metab (2016) 310(11):E994–1002. 10.1152/ajpendo.00121.2016 27166282 PMC4935144

[67] Aoki M Fujishita T. Oncogenic Roles of the PI3K/AKT/mTOR Axis. Curr Top Microbiol Immunol (2017) 407:153–89. 10.1007/82_2017_6 28550454

[68] Jais A Brüning JC. Hypothalamic inflammation in obesity and metabolic disease. J Clin Invest (2017) 127(1):24–32. 10.1172/JCI88878 28045396 PMC5199695

[69] Bartoszewska S Collawn JF. Unfolded protein response (UPR) integrated signaling networks determine cell fate during hypoxia. Cell Mol Biol Lett (2020) 25:18. 10.1186/s11658-020-00212-1 32190062 PMC7071609

[70] Xia Z Wu S Wei X Liao Y Yi P Liu Y. Hypoxic ER stress suppresses β-catenin expression and promotes cooperation between the transcription factors XBP1 and HIF1α for cell survival. J Biol Chem (2019) 294(37):13811–21. 10.1074/jbc.RA119.008353 PMC6746444 31350332

[71] Sakurai T Amemiya A Ishii M Matsuzaki I Chemelli RM Tanaka H. Orexins and Orexin Receptors: A Family of Hypothalamic Neuropeptides and G Protein-Coupled Receptors that Regulate Feeding Behavior. Cell (1998) 92(4):573–85. 10.1016/S0092-8674(00)80949-6 9491897

[72] Thannickal TC Moore RY Nienhuis R Ramanathan L Gulyani S Aldrich M. Reduced number of hypocretin neurons in human narcolepsy. Neuron (2000) 27(3):469–74. 10.1016/S0896-6273(00)00058-1 PMC8760623 11055430

[73] Ettrup KS Sørensen JC Bjarkam CR. The anatomy of the Göttingen minipig hypothalamus. J Chem Neuroanat (2010) 39(3):151–65. 10.1016/j.jchemneu.2009.12.004 20043984

[74] Gotter AL Webber AL Coleman PJ Renger JJ Winrow CJ International Union of Basic and Clinical Pharmacology. LXXXVI. Orexin Receptor Function, Nomenclature and Pharmacology. Pharmacol Rev (2012) 64(3):389–420. 10.1124/pr.111.005546 22759794

[75] Alexandre C Andermann ML Scammell TE. Control of arousal by the orexin neurons. Curr Opin Neurobiol (2013) 23(5):752–9. 10.1016/j.conb.2013.04.008 PMC3783629 23683477

[76] Sakurai T. The neural circuit of orexin (hypocretin): maintaining sleep and wakefulness. Nat Rev Neurosci (2007) 8(3):171–81. 10.1038/nrn2092 17299454

[77] Sikder D Kodadek T. The neurohormone orexin stimulates hypoxia-inducible factor-1 activity. Gene Dev (2007) 21(22):2995–3005. 10.1101/gad.1584307 18006690 PMC2049199

[78] Butterick TA Billington CJ Kotz CM Nixon JP. Orexin: Pathways to obesity resistance? Rev Endocr Metab Disord (2013) 14(4):357–64. 10.1007/s11154-013-9259-3 PMC4739824 24005942

[79] Garland TJ Schutz H Chappell MA Keeney BK Meek TH Copes LE. The biological control of voluntary exercise, spontaneous physical activity and daily energy expenditure in relation to obesity: human and rodent perspectives. J Exp Biol (2011) 214(Pt 2):206–29. 10.1242/jeb.048397 PMC3008631 21177942

[80] O’Hagan KA Cocchiglia S Zhdanov AV Tambuwala MM Cummins EP Monfared M. PGC-1alpha is coupled to HIF-1alpha-dependent gene expression by increasing mitochondrial oxygen consumption in skeletal muscle cells. Proc Natl Acad Sci USA (2009) 106(7):2188–93. 10.1073/pnas.0808801106 PMC2632715 19179292

